# Buckling behaviour of rectangular and skew plates with elastically restrained edges under non-uniform mechanical edge loading

**DOI:** 10.1371/journal.pone.0308245

**Published:** 2024-09-06

**Authors:** Chen Wang, Qiang Liu

**Affiliations:** AVIC General Huanan Aircraft Industry Co., Ltd, Zhuhai, China; Universiti Teknologi Malaysia, MALAYSIA

## Abstract

In this paper, the buckling behaviour of rectangular and skew plates with elastically restrained edges subjected to non-uniform mechanical edge loading is investigated. An analysis method is developed for calculating the critical buckling load of plates using the Ritz method under non-uniform mechanical edge loading, in which the shape function is expressed as Legendre polynomials. The in-plane stress distribution under non-uniform mechanical edge loading is defined by the pre-buckling analysis. Contributions of elastic boundary conditions are taken into accounted by giving different edge spring stiffnesses. The proposed method for buckling analysis of plates is validated by the comparison of exiting results in literature. Finally, the effects of the edge restrained stiffness, non-uniform edge loading, skew angle, aspect ratio and combined compression-shear load are discussed by parametric analysis.

## Introduction

In recent years, skew plates and stiffened plates have been widely and effectively used in transportation, aerospace, mechanical, and structural engineers. The analysis of bending, buckling and post-buckling of skew plates has important theoretical and practical significance, particularly the buckling behaviour of plates under non-uniform edge loads. Usually, plates are part of complex structural system, so the loads acting on the plates may not always be uniform. For instance, in the case of I-beam or wide flanged beam subjected to bending moment at the ends or lateral loads on the flange, the web of the beam is under non-uniform in-plane loads. The load applied by adjacent structures on the wings or stiffened plates in ship structures is usually nonuniform. Many researchers have done a lot of work in this field, however due to the non-orthogonality of the skew plates. It is difficult to carry out buckling analysis of skew plates under the special boundary conditions. Previous studies on the buckling behaviour of skew plates mainly focused on finite element analysis. Therefore, it is of great practical value to analyse the buckling behaviour of skew plates by using analytic method. The simply and clamped supported boundary conditions are two ideal boundary conditions. In reality, the boundary conditions between joined structures are usually elastically restrained edges. The buckling behaviour of skew plates with elastically restrained edges under non-uniform mechanical edge loading has been rarely studied in the literature.

In the previous studies, the finite element analysis (FEA), differential quadrature method (DQM) and Ritz energy method were mainly used to analyse the buckling behaviour of plates. Additionally, with the rapid development of computer technology, the finite element method was widely used in the buckling analysis of plates. The buckling behaviour of skew composite laminate plates subjected to uniaxial edge loading was studied using the ABAQUS by Hu and Tzeng [1]. Thermal buckling of a skew functionally graded (FG) plate with four edges simply supported was is investigated by the FEA, including the effect of first-order shear deformation theory by Ganapathi and Prakash [2]. M Khorasani et al. [3] assessed its thermo-elastic buckling behavior of a micro-scaled sandwich rectangular plate using the Halpin-Tsai and extended rule of mixture (ERM) schemes. Prakash et al. [4] investigated the post-buckling behaviour of functionally graded material (FGM) skew plates that are under a thermal load by the FEA. The buckling loads of skew laminate plates with variable thickness were obtained through the ANSYS by Dhurvey and Priyanka [5]. The vibration and buckling analyses of skew plates with edges elastically restrained against rotation were discussed with the spline finite strip method (FSM) by Mizusawa and Kajita [6]. The post-buckling of fiber-reinforced plastic (FRP) composite structural shapes with elastically restrained edges under end shortening was investigated with the FSM by Qiao and Chen [7]. Y. Kiani et al. [8] investigated the free vibration of a skew cylindrical shell using the Chebyshev-Ritz formulation, in which the cylindrical panel was made of functionally graded carbon nanotube reinforced composites. Y Kiani and KK Zur [9] investigated the free vibrations of composite laminated skew plates by general idea of Ritz method where the shape functions were constructed with the aid of Chebychev polynomials. Junsheng Zhu et al. [10] performed a free vibration investigation on skew sandwich plates with functionally graded metal foam core using the general idea of Ritz method. R. Jahanbazi et al. [11] performed a free vibration investigation on composite laminated skew cylindrical shells reinforced with graphene platelets using the general idea of Ritz method.

For rapid analysis of the buckling and post-buckling behaviour of plates, many analytical methods have been proposed over the last few decades based on several simplified assumptions. The natural frequencies of skew FG-CNTRC plates were obtained using the Ritz method, in which shape functions are defined with the Gram-Schmidt process by Kiani [12]. Y. Kiani [13] employed the non-uniform rational B-spline iso-geometric finite element formulation to study the thermal buckling behaviour of composite laminated skew plates based on the first-order shear deformation plate theory. The buckling behaviour of laminated composite skew plates under linearly varying edge loading was obtained using a Rayleigh-Ritz method with the Gram-Schmidt process by Kumar et al. [14]. Y Kiani [15] studied the buckling behaviour of FG-CNT-reinforced composite plates under parabolic loading using the Ritz method and Airy stress function formulation. Critical buckling temperatures of FG- CNTRC skew plates were obtained with the Gram–Schmidt process by Kiani and Yaser [16]. The buckling load of thin skew fibre-reinforced composite laminates was solved by a B-spline Rayleigh-Ritz method (RRM) based on classical plate theory (CPT) [17–19]. The geometrically nonlinear large deformation analysis of FG- CNTRC skew plates on Pasternak foundations was first presented using the element-free IMLS-Ritz method by Zhang and Liew [20]. The vibration analyses of FG-CNTRC skew plates were carried out with the Ritz procedure, including the effect of transverse shear deformation by Zhang et al. [21]. The buckling loads of FG-CNTRC thick skew plates on Pasternak foundations were obtained with the element-free IMLS-Ritz method, which sufficiently considers the effects of transverse shear deformation and rotary inertia by Lei et al. [22]. The mechanical behaviour of laminated CNT-reinforced composite skew plates subjected to a transverse sudden dynamic load was first presented using the element-free IMLS-Ritz method by Zhang [23]. Free vibration analysis of isotropic skew plates was carried out using the spectral collocation method by Mohazzab and Dozio [24].

The critical buckling loads and vibration frequency of rectangular and skew plates under simply supported or clamped boundary conditions were obtained with differential quadrature method (DQM) by Wang et al. [25, 26]. The buckling, bending and vibration behaviour of CNT-reinforced composite skew plates based on Reddy’s higher order shear deformation theory (HSDT) was studied with the isogeometric method by Zhang and Memar [27, 28]. The buckling analysis of rectangular composite plates subjected to non-uniform edge loading was studied under nine sets of different boundary conditions by Panda and Ramachandra [29]. The buckling, post-buckling and post-buckled vibration behaviour of composite skew plates subjected to non-uniform in-plane loadings are presented, which sufficiently considers the effects of initial geometric imperfections by Kumar et al. [30]. The non-linear dynamic instability of damped composite skew plates under non-uniform in-plane periodic loadings was studied using analytical methods by Kumar et al. [31]. The dynamic instability analysis of a functionally graded skew plate subjected to uniform and linearly varying periodic edge loadings was carried out using Gram–Schmidt process under different boundary conditions by Kumar et al. [32]. Kiani and Mirzaei [33] studied the shear buckling behaviour of composite skew plates under two different types of edge loading was studied by using Gram–Schmidt process. Civalek O and Jalaei M H [34] investigated the shear buckling behaviour of functionally graded (FG) carbon nanotube reinforced skew plates under different boundary conditions using a four-nodded straight-sided geometric element.

In recent years, the buckling behaviour of plates under uniform edge loading are studied using various numerical approaches. However, very few researchers have focused thus far on the buckling behaviour of skew plates with elastically restrained edges under non-uniform mechanical edge loading. The main objective of the present work is to present an analysis method for the buckling behaviour of rectangular and skew plates with elastically restrained edges under non-uniform mechanical edge loading, in which the shape function is expressed as Legendre polynomials. The proposed method is proved the validity and accuracy by comparison with the previous literatures. Parametric analysis is made to examine the effects of the edge restrained stiffness, non-uniform edge loading, skew angle, aspect ratio and compression-shear load on the buckling behaviour of rectangular and skew plates.

## Theoretical formulation

An analytical method for the buckling behaviour of plates with elastically restrained edges subjected to non-uniform mechanical edge loading is developed in this paper. The stress distribution within the plates under non-uniform mechanical edge loading is obtained by solving the minimum strain energy using the Ritz method. Simple closed-form approximations in the form of combinations of Legendre polynomials are employed for the description of plate deformation. The governing equations of the elastically restrained plate under non-uniform mechanical edge loading are solved by the Ritz method.

**Fig 1:**
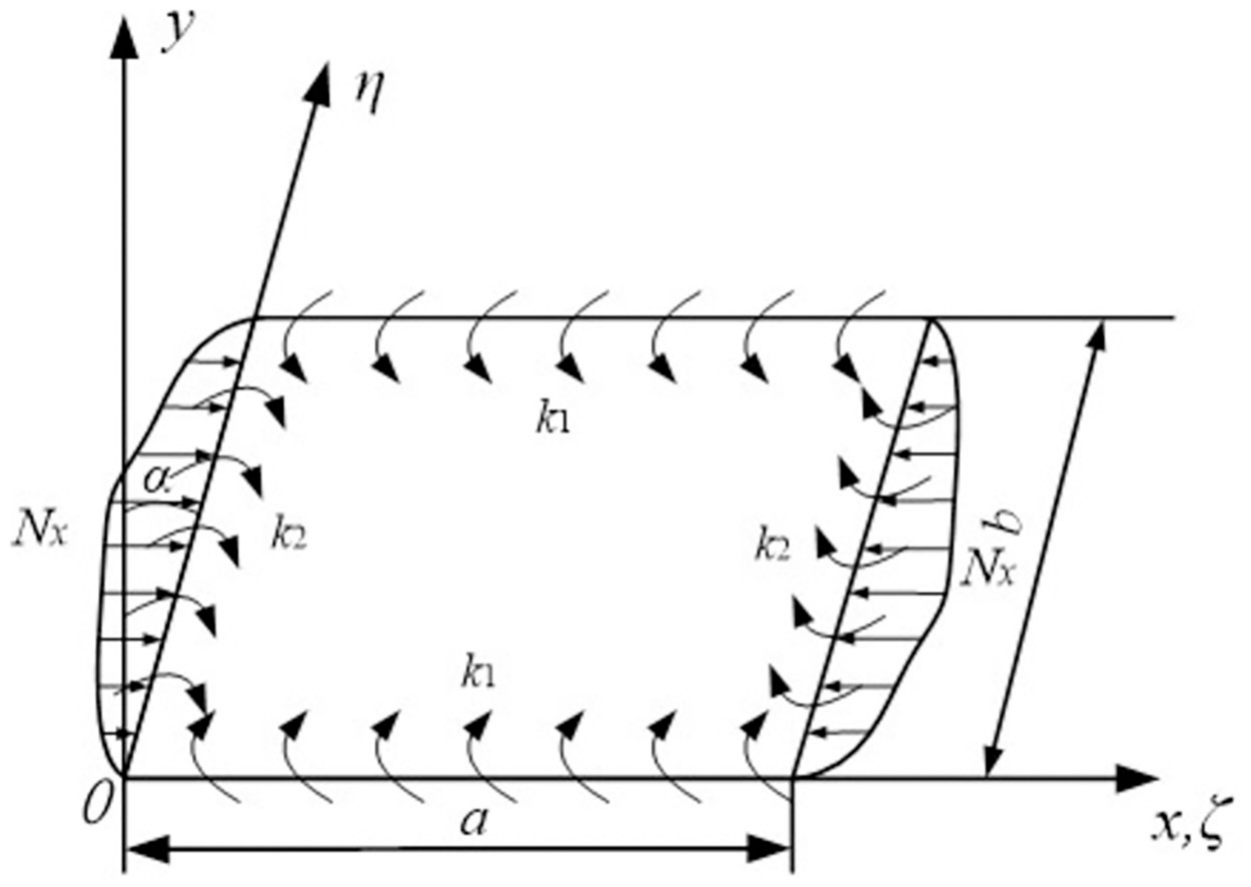
Geometry feature of the skew plate under non-uniform mechanical edge loading.

The plate under Cartesian coordinate system is shown in Fig 1, in which the plate with length *a*, breadth *b* and thickness *h* is under in-plane combined loads *N*_*x*_ and *N*_*xy*_. The longitudinal and transverse edge spring stiffnesses are denoted as *k*_1_ and *k*_2_, respectively. The skew angle is denoted as *α*. The theoretical model for the buckling analysis of plate with elastically restrained edges is established in the displacement field. The displacement components of the plate along *x*, *y* and *z* directions can be expressed based on first-order shear deformation (FSDT) theory:

$$\begin{array}{c} u(x,y,z)=u_{0}(x,y)-z\frac{\partial w}{\partial x}\\ v(x,y,z)=v_{0}(x,y)-z\frac{\partial w}{\partial y}\\ w(x,y,z)=w(x,y) \end{array}$$ (1)

Where *u*, *v* and *w* are the displacement components of any point at the mid-plane of the plate along x, y and z directions; features of the mid-plane of the plate are indicated by a subscript 0. The corresponding strain components can be written as follows:

$$\left\{\begin{array}{l} \varepsilon_{x}\\ \varepsilon_{y}\\ \gamma_{xy} \end{array}\right\}=\left\{\begin{array}{l} \varepsilon_{x}^{0}\\ \varepsilon_{y}^{0}\\ \gamma_{xy}^{0} \end{array}\right\}+z\left\{\begin{array}{l} \psi_{x}\\ \psi_{y}\\ \psi_{xy} \end{array}\right\}$$ (2)

Where the features of the mid-plane of the plate are again indicated by a subscript 0.

According to linear elastic theory, the constitutive relations of the plate can be written as:

$$\left\{\begin{array}{l} \sigma_{x}\\ \sigma_{y}\\ \tau_{xy} \end{array}\right\}=\left[\begin{array}{ccc} Q_{11} & Q_{12} & 0\\ Q_{11} & Q_{12} & 0\\ 0 & 0 & Q_{66} \end{array}\right]\left\{\begin{array}{l} \varepsilon_{x}\\ \varepsilon_{y}\\ \gamma_{xy} \end{array}\right\}$$ (3)

The force and moment resultants of the plate can be obtained by integrating the stress along the thickness of the plate as follows:

$$\left(\left(\begin{array}{l} N_{x}\\ N_{y}\\ N_{xy} \end{array}\right)\;\left(\begin{array}{l} M_{x}\\ M_{y}\\ M_{xy} \end{array}\right)\right)=\int_{-h/2}^{h/2}\left(\begin{array}{l} \sigma_{x}\\ \sigma_{y}\\ \tau_{xy} \end{array}\right)\;\left(1,z\right)dz$$ (4)

The force and moment resultants can then be obtained by integration of Eqs (2), (3) and (4):

$$\left\{\begin{array}{l} \left\{N\right\}\\ \left\{M\right\} \end{array}\right\}=\left[\begin{array}{ll} \left[A\right] & \left[B\right]\\ \left[B\right] & \left[D\right] \end{array}\right]\;\left\{\begin{array}{l} \left\{\varepsilon^{0}\right\}\\ \left\{\psi \right\} \end{array}\right\}$$ (5)

Where [*A*] is the extensional stiffness matrix, [*B*] is the coupling stiffness matrix, and [*D*] is the bending stiffness matrix. The tension–bending coupling stiffness matrix of the plate is zero.

$$\begin{array}{c} \left\{\varepsilon_{0}\right\}=\left[\varepsilon_{x}^{0},\varepsilon_{y}^{0},\gamma_{xy}^{0}\right]\;\left\{\psi \right\}=\left[\psi_{x},\psi_{y},\psi_{xy}\right]\\ \left\{N\right\}=\left[N_{x},N_{y},N_{xy}\right]\;\left\{M\right\}=\left\{M_{x},M_{y},M_{xy}\right\}\\ \left[A\right]=\left[\begin{array}{lll} A_{11} & A_{12} & A_{16}\\ A_{21} & A_{22} & A_{26}\\ A_{16} & A_{26} & A_{66} \end{array}\right]\;\left[D\right]=\left[\begin{array}{lll} D_{11} & D_{12} & D_{16}\\ D_{21} & D_{22} & D_{26}\\ D_{16} & D_{26} & D_{66} \end{array}\right] \end{array}$$ (6)

Where *N*_*x*_, *N*_*y*_ and *N*_*xy*_ are the force resultants; *M*_*x*_, *M*_*y*_ and *M*_*xy*_ are moment resultants;$\varepsilon_{x}^{0}$, $\varepsilon_{y}^{0}$ and $\varepsilon_{xy}^{0}$ are the normal and shear strains; *ψ*_*x*_, *ψ*_*y*_ and *ψ*_*xy*_ are curvatures of the plate.

For the skew plate, any point (*x*, *y*) in oblique co-ordinate system can be transformed to (*ξ*, *η*) orthogonal co-ordinate system based on the coordinate transformation. The relationship between orthogonal co-ordinate system and oblique co-ordinate system can be expressed as:

$$\begin{array}{c} \xi =x-y\;\text{tan}\;\alpha \\ \eta =y\;\text{sec}\;\alpha \end{array}$$ (7)

Then the first order derivatives in the relationship of two coordinate systems are defined as

$$\begin{array}{c} (){,}_{x}=(){,}_{\xi }\\ \left(){,}_{y}=-(){,}_{\xi }\;\text{tan}(\alpha )+(){,}_{\eta }\;\text{sec}(\alpha \right) \end{array}$$ (8)

The second order derivatives in the relationship of two coordinate systems are defined as

$$\begin{array}{l} (){,}_{xx}=(){,}_{\xi \xi }\\ (){,}_{yy}={\text{tan}}^{2}(\alpha )(){,}_{\xi \xi }+{\text{sec}}^{2}(\alpha )(){,}_{\eta \eta }-2\;\text{tan}(\alpha )\;\text{sec}(\alpha )(){,}_{\xi \eta }\\ (){,}_{xy}=-\text{tan}(\alpha )(){,}_{\xi \xi }+\text{sec}(\alpha )(){,}_{\xi \eta } \end{array}$$ (9)

Where *α* is the skew angle of the plate, *ξ* and *η* are the coordinate variables of oblique co-ordinate system.

For the buckling behaviour of skew plates, the equilibrium equation of the plate with elastically restrained edges is established using the minimal potential energy method. The equilibrium equation of the plate can be written as follows:

$$\delta (U+U_{k}-W)=0$$ (10)

Where *U*, *U*_*k*_ and *W* indicate the potential strain energy, the potential energy stored in the edge springs and the work done by edge loadings. The potential strain energy of the plate in the equilibrium equation is given as follows:

$$U=\int_{A}\int_{-h/2}^{h/2}(\sigma_{x}\varepsilon_{x}+\sigma_{y}\varepsilon_{y}+\tau_{xy}\gamma_{xy})dAdz$$ (11)

For the elastically restrained plate, the potential energy of the edge springs in the equilibrium equation is given by the following:

$$\begin{array}{c} U_{k}=\frac{1}{2}k_{1}\int_{-a}^{a}w_{,y}\delta w_{,y}{|}_{y=-b}dx+\frac{1}{2}k_{1}\int_{-a}^{a}w_{,y}\delta w_{,y}{|}_{y=b}dx+\\ \frac{1}{2}k_{2}\int_{-b}^{b}w_{,x}\delta w_{,x}{|}_{x=-a}dy+\frac{1}{2}k_{2}\int_{-b}^{b}w_{,x}\delta w_{,x}{|}_{x=a}dy \end{array}$$ (12)

For the elastically restrained plate, the work done by the edge loading the equilibrium equation is given as follows:

$$W=\int_{A}(N_{x}^{0}w_{,x}\delta w_{,x}+N_{y}^{0}w_{,y}\delta w_{,y}+N_{xy}^{0}w_{,x}\delta w_{,y}+N_{xy}^{0}w_{,y}\delta w_{,x})dA$$ (13)

## Solution procedure

### The stress distribution function

In this study, the buckling behaviour of elastically restrained skew plates under different non-uniform mechanical edge loadings is investigated, in which the in-plane stress distribution is defined by the pre-buckling analysis. In general, the stress within the plate under non-uniform edge loading is distributed differently along three directions. The stress distribution function of the plate under non-uniform edge loading can be calculated by principle of minimum strain energy. The membrane strain energy of the skew plate is given as follows:

$$V=\frac{1}{2}{\iint_{\bar{A}}\left\{\begin{array}{l} n_{\xi \xi }\\ n_{\eta \eta }\\ n_{\xi \eta } \end{array}\right\}}^{T}{\left[\begin{array}{lll} {\bar{A}}_{11} & {\bar{A}}_{12} & {\bar{A}}_{16}\\ {\bar{A}}_{21} & {\bar{A}}_{22} & {\bar{A}}_{26}\\ {\bar{A}}_{16} & {\bar{A}}_{26} & {\bar{A}}_{66} \end{array}\right]}^{-1}\left\{\begin{array}{l} n_{\xi \xi }\\ n_{\eta \eta }\\ n_{\xi \eta } \end{array}\right\}\;\text{cos}\;\alpha d\xi d\eta$$ (14)

Where

$$n_{\xi \xi }=\frac{\partial^{2}\phi }{\partial \eta^{2}},n_{\eta \eta }=\frac{\partial^{2}\phi }{\partial \xi^{2}},n_{\xi \eta }=-\frac{\partial^{2}\phi }{\partial \xi \partial \eta }$$ (15)

Besides, the components of the stress resultants in orthogonal co-ordinate system can be expressed as [35]:

$$\begin{array}{l} n_{xx}=n_{\xi \xi }+n_{\eta \eta }\;{\text{sin}}^{2}\;\alpha +2n_{\xi \eta }\;\text{sin}\;\alpha \\ n_{yy}=n_{\eta \eta }\;{\text{cos}}^{2}\;\alpha \\ n_{xy}=n_{\eta \eta }\;\text{sin}\;\alpha \;\text{cos}\;\alpha +n_{\xi \eta }\;\text{cos}\;\alpha \end{array}$$ (16)

The extensional stiffness $\bar{A}$ in oblique co-ordinate system can be solved by using the coordinate transformation matrix *T* with the extensional stiffness *A* in orthogonal co-ordinate system.

$$\bar{A}=T^{-T}AT^{-1}$$ (17)

Where

$$T=\left[\begin{array}{lll} 1 & 0 & 0\\ {\text{sin}}^{2}\;\alpha & {\text{cos}}^{2}\;\alpha & \text{sin}\;\alpha \;\text{cos}\;\alpha \\ 2\;\text{sin}\;\alpha & 0 & \text{cos}\;\alpha \end{array}\right]$$ (18)

Where *ϕ* is the in-plane stress function. Based on the minimum strain energy, the stress function is determined by the Ritz method. The stress function in the form of a series of polynomials is written as:

$$\phi =\phi_{0}+\beta_{1}\phi_{1}+\beta_{2}\phi_{2}+\beta_{3}\phi_{4}+\ldots$$ (19)

Where the mechanical non-uniform edge loading is expressed as *ϕ*_0_. Therefore, the stress function is defined as follows:

$$\phi =\phi_{0}+{\left({\left(\frac{a}{x}\right)}^{2}-1\right)}^{2}{\left({\left(\frac{b}{y}\right)}^{2}-1\right)}^{2}\left(\beta_{1}+\beta_{2}x+\beta_{3}y+\beta_{4}xy+\beta_{5}x^{2}+\beta_{6}y^{2}\right)$$ (20)

It is assumed that the resultant forces resulting from the distributed edge loading at two opposite edges are equal and the distributed edge loads are axisymmetric with respect to the *y* axis. The resultant forces resulting from the distributed edge loading at two opposite edges are defined as follows:

$$\int_{0}^{\text{b}}\frac{\partial^{2}\phi_{0}}{\partial x^{2}}dy=\text{constant}$$ (21)

**Fig 2:**
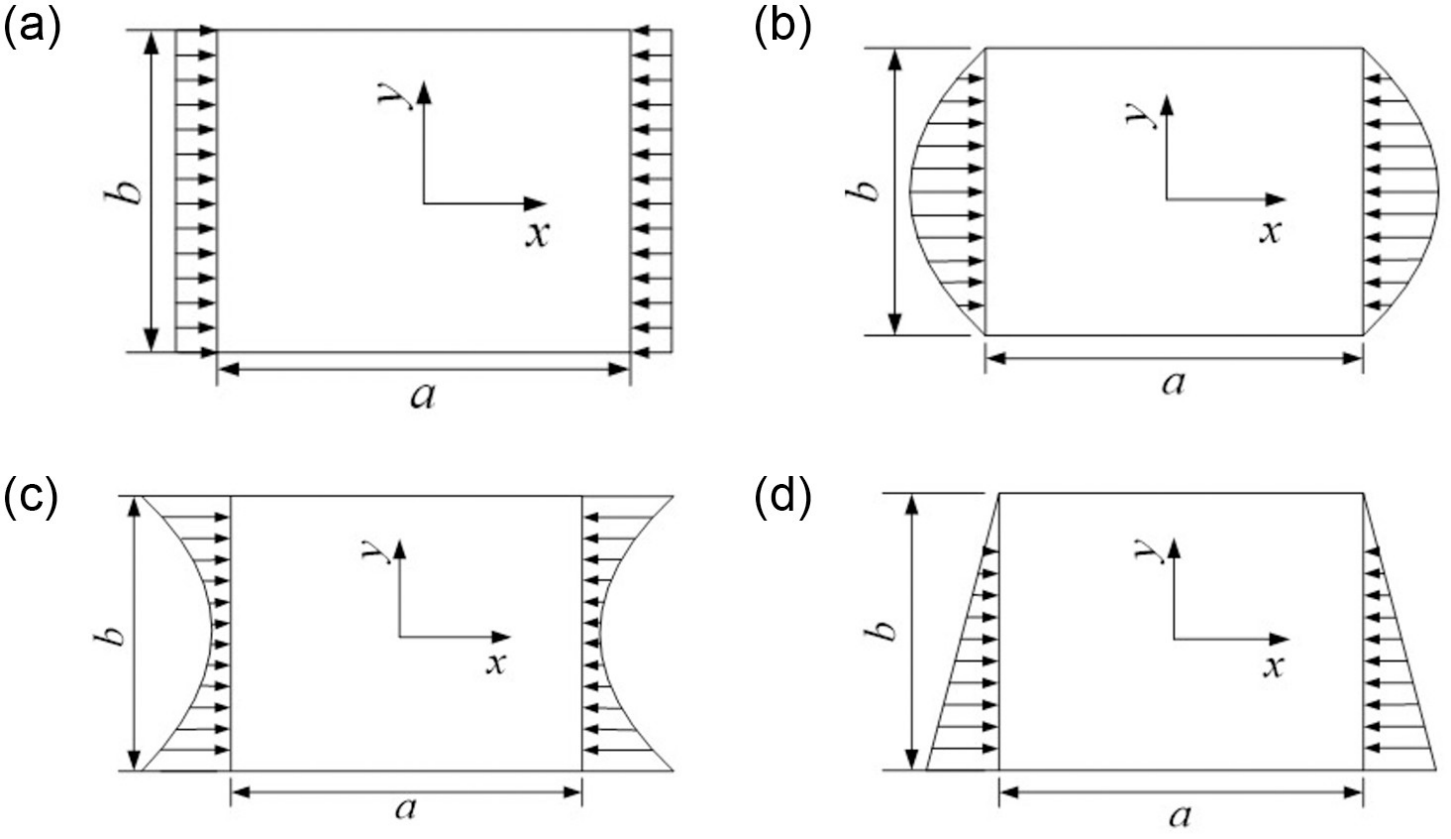
Cases of non-uniform mechanical edge loading. (a). Uniform loading. (b). Parabolic loading (convex function). (c). Parabolic loading (concave function). (d). Trapezoidal loading.

For uniform, parabolic (convex function), parabolic (concave function) and trapezoidal edge loading, the stress distribution function of the plate is solved by the Ritz method, in which the number of series in the stress function is 8. The four different edge loadings are considered in Fig 2.

#### Uniform edge loading

The boundary conditions for the plate under uniform mechanical edge loading are given as follows:

$$\begin{array}{c} x=0,a\;{\bar{N}}_{x}=\frac{2}{3}N_{0}\;{\bar{N}}_{xy}=0\\ y=0,b\;{\bar{N}}_{y}=0\;{\bar{N}}_{xy}=0 \end{array}$$ (22)

The stress function of the plate under uniform mechanical edge loading is written as follows:

$$\phi =\frac{1}{3}N_{0}y^{2}+{\left({\left(\frac{x}{a}\right)}^{2}-1\right)}^{2}{\left({\left(\frac{y}{b}\right)}^{2}-1\right)}^{2}\left(\beta_{1}+\beta_{2}x+\beta_{3}y+\beta_{4}xy+\beta_{5}x^{2}+\beta_{6}y^{2}\right)$$ (23)

#### Parabolic edge loading (convex function)

The boundary conditions for the plate under parabolic mechanical edge loading (convex function) are given as follows:

$$\begin{array}{c} x=0,a\;{\bar{N}}_{x}=N_{0}(1-\frac{y^{2}}{b^{2}})\;{\bar{N}}_{xy}=0\\ y=0,b\;{\bar{N}}_{y}=0\;{\bar{N}}_{xy}=0 \end{array}$$ (24)

The stress function within the plate under parabolic mechanical edge loading is written as follows:

$$\phi =N_{0}(\frac{1}{2}y^{2}-\frac{1}{12}\frac{y^{4}}{b^{2}})+{\left({\left(\frac{x}{a}\right)}^{2}-1\right)}^{2}{\left({\left(\frac{y}{b}\right)}^{2}-1\right)}^{2}\left(\beta_{1}+\beta_{2}x+\beta_{3}y+\beta_{4}xy+\beta_{5}x^{2}+\beta_{6}y^{2}\right)$$ (25)

#### Parabolic edge loading (concave function)

The boundary conditions for the plate under parabolic mechanical edge loading (concave function) are given as follows:

$$\begin{array}{c} x=0,a\;{\bar{N}}_{x}=N_{0}(\frac{1}{2}+\frac{5y^{4}}{6b^{4}})\;{\bar{N}}_{xy}=0\\ y=0,b\;{\bar{N}}_{y}=0\;{\bar{N}}_{xy}=0 \end{array}$$ (26)

The stress function of the plate under parabolic mechanical edge loading is written as follows:

$$\phi =N_{0}(\frac{1}{4}y^{2}+\frac{y^{6}}{36b^{4}})+{\left({\left(\frac{x}{a}\right)}^{2}-1\right)}^{2}{\left({\left(\frac{y}{b}\right)}^{2}-1\right)}^{2}\left(\beta_{1}+\beta_{2}x+\beta_{3}y+\beta_{4}xy+\beta_{5}x^{2}+\beta_{6}y^{2}\right)$$ (27)

#### Trapezoidal edge loading

The boundary conditions for the plate under trapezoidal mechanical edge loading are given as follows:

$$\begin{array}{c} x=0,a\;{\bar{N}}_{x}=N_{0}\frac{2}{3}\left(\frac{y}{b}+1\right)\;{\bar{N}}_{xy}=0\\ y=0,b\;{\bar{N}}_{y}=0\;{\bar{N}}_{xy}=0 \end{array}$$ (28)

The stress function of the plate under trapezoidal mechanical edge loading is written as follows:

$$\phi =-N_{0}\frac{2}{3}\left(\frac{1}{6}\frac{{\left(y\right)}^{3}}{b}-\frac{1}{2}y^{2}\right)+{\left({\left(\frac{x}{a}\right)}^{2}-1\right)}^{2}{\left({\left(\frac{y}{b}\right)}^{2}-1\right)}^{2}\left(\beta_{1}+\beta_{2}x+\beta_{3}y+\beta_{4}xy+\beta_{5}x^{2}+\beta_{6}y^{2}\right)$$ (29)

The constants *β*_*i*_ can be obtained by principle of minimum strain energy while the stress function needs to satisfy the boundary conditions.

### Legendre polynomials and the Ritz method

The governing equilibrium equations of the plate can be derived based on principle of minimum potential energy. The resulting equations should be solved to obtain the critical buckling loads of plates with elastically restrained edges under non-uniform mechanical edge loading. The shape function which satisfies boundary conditions is used to describe plate deformation along *x*, *y* and *z* directions. In this paper, the transverse shape function is defined as the combination of Legendre polynomials and auxiliary functions as follows:

$$\begin{array}{l} u(x,y)=F_{u}(x,y)\sum_{i=1}^{N_{x}}\;\sum_{j=1}^{N_{y}}\;u_{ij}P_{i}(x)P_{j}(y)\\ v(x,y)=F_{v}(x,y)\sum_{i=1}^{N_{x}}\;\sum_{j=1}^{N_{y}}\;v_{ij}P_{i}(x)P_{j}(y)\\ w(x,y)=F_{w}(x,y)\sum_{i=1}^{N_{x}}\;\sum_{j=1}^{N_{y}}\;w_{ij}P_{i}(x)P_{j}(y) \end{array}$$ (30)

Where *p*_*i*_(*x*) and *p*_*j*_(*y*) are Legendre polynomials of order *i* and *j*, respectively; *u*_*ij*_, *v*_*ij*_ and *w*_*ij*_ are coefficients relative to *i* and *j* order Legendre polynomials along *x*, *y* and *z* directions.

When *n* is an integer, the Legendre polynomial that satisfies the boundary conditions at *x* = ±1 is defined as:

$$P_{n}(x)=\sum_{m=0}^{M}\frac{{\left(-1\right)}^{m}(2n-2m)!}{2^{n}\cdot m!(n-m)!(n-2m)!}x^{n-2m},\;-1<x<1$$

where

$$M=\left\{\begin{array}{ll} \frac{n}{2}, & n:\text{even}\;\text{number}\\ \frac{n-1}{2}, & n:odd\;number \end{array}\right.$$ (31)

*F*_*u*_(*x*, *y*), *F*_*v*_(*x*, *y*) and *F*_*w*_(*x*, *y*) are the auxiliary functions. In order to satisfy the corresponding boundary conditions, the auxiliary functions are defined as follows:

$$\begin{array}{l} F_{u}(x,y)={\left(\frac{x}{a}+1\right)}^{s_{1}}{\left(\frac{x}{a}-1\right)}^{s_{2}}\\ F_{v}(x,y)={\left(\frac{y}{b}+1\right)}^{s_{3}}{\left(\frac{y}{b}-1\right)}^{s_{4}}\\ F_{w}(x,y)={\left(\frac{x}{a}+1\right)}^{s_{1}}{\left(\frac{x}{a}-1\right)}^{s_{2}}{\left(\frac{y}{b}+1\right)}^{s_{3}}{\left(\frac{y}{b}-1\right)}^{s_{4}} \end{array}$$ (32)

The exponents *s*_1_, *s*_2_, *s*_3_ and *s*_4_ can be determined according to the corresponding boundary conditions and are equal to 0, 1 or 2. The exponential values under relatively different boundary conditions are listed in Table 1. *S* denotes simply supported boundary, *C* denotes clamped supported boundary.

**Table 1:** Exponents in the auxiliary function for different boundary conditions (BC).

BC	*S*_*1*_	*S*_*2*_	*S*_*3*_	*S*_*4*_
S-S	1	1	1	1
C-C	2	2	2	2
S-C	1	1	2	2

The skew plate with elastically restrained edges is considered in this paper. The edge spring stiffness *k*_*α*_ is introduced for buckling analysis of plates under non-uniform mechanical edge loading in this paper. The clamped and simply supported boundary conditions can be changed by adjusting the exponential values *k*_*α*_. When the edge spring stiffness *k*_*α*_ is zero, the boundary conditions of the plate can be treated as simply supported. Conversely, when the edge spring stiffness *k*_*α*_ is infinitely large, the boundary conditions of the plate can be treated as clamped supported [36].

By introducing the shape function into Eq (30) into the equilibrium expression in Eq (10), the eigenvalue equation for the buckling behaviour of plates under non-uniform mechanical edge loading is written as follows:

$$(K-\lambda K_{g})X=0$$ (33)

Where *K* is the elastic stiffness matrix; *K*_*g*_ is the geometric stiffness matrix; *λ* is the critical buckling load; *X* is the unknown displacement vector of the plate about the unknown *u*, *v* and *w*.

## Numerical results and discussion

The buckling behaviour of plates with elastically restrained edges under non-uniform mechanical edge loading is studied in the paper. The proposed method is proved the validity and accuracy by comparison with the literatures.$K_{x0}^{r}$, $K_{xa}^{r}$, $K_{y0}^{r}$ and $K_{yb}^{r}$ are the dimensionless edge restrained spring stiffness coefficients along all edges. The dimensionless edge restrained spring stiffness coefficients are defined as follows [36]:

$$\begin{array}{l} K_{x0}^{r}=\frac{k_{2}b}{\sqrt{D_{11}D_{22}}}\;K_{xa}^{r}=\frac{k_{2}b}{\sqrt{D_{11}D_{22}}}\\ K_{y0}^{r}=\frac{k_{1}a}{\sqrt{D_{11}D_{22}}}\;K_{yb}^{r}=\frac{k_{1}a}{\sqrt{D_{11}D_{22}}} \end{array}$$ (34)

For the convenience of the study, the dimensionless longitudinal and transverse edge spring stiffness coefficients are set to $K_{x0}^{r}=K_{xa}^{r}=K_{2}$$K_{y0}^{r}=K_{yb}^{r}=K_{1}$. The dimensionless edge spring stiffness coefficients *K*_1_ = *K*_2_ = 10^4^ along all edges can be treated as the clamped support. These extreme boundary conditions are proved based on the following discussions.

### Convergence and comparison studies

In this section, the convergence is studied to prove the efficiency and accuracy of the analysis method by changing the number of terms of Legendre polynomials in the trial function. A plate with elastically restrained edges under uniform and non-uniform mechanical edge loading is considered. The geometric parameters and material properties of the aluminium plate are as follows: modulus of elasticity *E* = 70 GPa, Poisson’s ratio *v* = 0.3, aspect ratio *a*/*b* = 1 and thickness *h* = 0.001 *m*. The results of the convergence analysis with different numbers of terms of Legendre polynomials in the trial function are compared with previously literature are summarised in Table 1. In following results, the dimensionless critical buckling load factor is defined as follows:

$$\lambda_{cr}=\frac{N_{cr}b^{2}}{\pi^{2}\sqrt{D_{11}D_{22}}}$$ (35)

Where *D*_11_ and *D*_22_ are the bending stiffnesses, *N*_*cr*_ is the critical buckling load. It can be seen that the calculated results listed in Tables 2–7 satisfy the accuracy requirements when the number of polynomial terms in the trial function is *N*_*x*_ = *N*_*y*_ = 8. Therefore, the number of series in shape function of Ritz method is 16. Further, it is observed that the calculation results of the edge restrained spring stiffness coefficients *K*_1_ = *K*_2_ = 0 and *K*_1_ = *K*_2_ = 10^4^ are in well agreement with buckling results of simply supported and clamped supported plates by Chen [36]. The accuracy and validity of the proposed method is demonstrated by the convergence and comparison study. The theoretical solution for critical buckling load of the plate is given as follows:

$$N_{cr}=\chi \frac{\pi^{2}D}{b^{2}}$$ (36)

**Table 2:** Convergence study of the longitudinal critical buckling loads of plates with elastically restrained edges.

	polynomial terms (*N*_*x*_ × *N*_*y*_)						
*K*	3×3	4×4	5×5	6×6	7×7	8×8	9×9
exact	4.000						
Chen [36]	3.989						
0 (S-S)	4.001	4.001	4.000	4.000	-	-	-
5	6.664	6.664	6.602	6.602	-	-	-
10	7.882	7.882	7.699	7.699	7.696	7.696	-
100	10.437	10.437	9.727	9.727	9.702	9.702	-
1000	10.888	10.888	10.066	10.066	10.034	10.034	-
10000	10.937	10.937	10.103	10.103	10.070	10.070	-
+∞(C-C)	10.942	10.942	10.107	10.107	10.074	10.074	-
exact	10.070						
Chen [36]	10.094						

**Table 3:** Convergence study of the critical shear buckling loads of plates with elastically restrained edges.

	polynomial terms (*N*_*x*_ × *N*_*y*_)						
*K*	3×3	4×4	5×5	6×6	7×7	8×8	9×9
exact	9.340						
Chen [28]	9.324						
0 (S-S)	10.944	9.832	9.338	9.328	9.325	9.324	9.324
5	15.238	12.188	11.264	11.225	11.217	11.216	11.216
10	18.295	13.341	12.120	12.062	12.050	12.048	12.048
100	45.724	16.868	14.282	14.173	14.134	14.127	14.127
1000	139.838	17.868	14.761	14.642	14.593	14.584	14.584
10000	440.657	17.990	14.816	14.695	14.646	14.636	14.636
+∞(C-C)		18.004	14.822	14.701	14.651	14.642	14.642
exact	14.710						
Chen [36]	14.718						

**Table 4:** Convergence study of the longitudinal critical buckling loads of plates with elastically restrained edges subjected to parabolic edge loading (convex function).

	polynomial terms (*N*_*x*_ × *N*_*y*_)						
*K*	3×3	4×4	5×5	6×6	7×7	8×8	9×9
Wang [37]	5.262						
0 (S-S)	5.248	5.248	5.244	5.244	-	-	-
5	8.880	8.880	8.843	8.843	-	-	-
10	10.564	10.564	10.383	10.383	10.382	-	-
100	14.078	14.078	13.112	13.112	13.086	13.086	-
1000	14.699	14.699	13.559	13.559	13.524	13.524	-
10000	14.766	14.766	13.607	13.607	13.571	13.571	-
+∞(C-C)	14.774	14.774	13.613	13.613	13.577	13.576	13.576
Wang [37]	13.580						

**Table 5:** Convergence study of the longitudinal critical buckling loads of plates with elastically restrained edges subjected to parabolic edge loading (concave function).

	polynomial terms (*N*_*x*_ × *N*_*y*_)						
*K*	3×3	4×4	5×5	6×6	7×7	8×8	9×9
FEM	6.562						
0 (S-S)	6.588	6.578	6.568	6.568	-	-	-
5	13.731	12.730	12.371	12.371	-	-	-
10	14.482	14.482	13.831	13.831	-	-	-
100	17.158	17.158	15.937	15.936	15.936		-
1000	17.531	17.531	16.226	16.225	16.167	16.167	-
10000	17.570	17.570	16.256	16.256	16.197	16.197	-
+∞(C-C)	16.259	16.259	16200	16.200	16.199	16.191	16.191
FEM	16.180						

**Table 6:** Convergence study of the longitudinal critical buckling loads of plates with elastically restrained edges subjected to trapezoidal loading.

	polynomial terms (*N*_*x*_ × *N*_*y*_)						
*K*	3×3	4×4	5×5	6×6	7×7	8×8	9×9
FEM	5.852						
0 (S-S)	5.905	5.861	5.860	5.859	5.859	-	-
5	11.667	11.464	11.154	11.151	11.144	11.144	-
10	13.354	13.078	12.449	12.445	12.425	12.425-	-
100	15.995	15.586	14.325	14.318	14.263	14.263	-
1000	16.370	15.942	14.590	14.582	14.521	14.521	-
10000	16.410	15.980	14.617	14.610	14.548	14.548	-
+∞(C-C)	14.621	14.613	14.551	14.551	14.550	14.550	
FEM	14.541						

**Table 7:** Convergence study of the longitudinal critical buckling loads of skew plates with elastically restrained edges *α* = 45^0^.

	polynomial terms (*N*_*x*_ × *N*_*y*_)						
*K*	3×3	4×4	5×5	6×6	7×7	8×8	9×9
Lei [22]	10.143						
0 (S-S)	12.986	11.572	10.752	10.445	10.304	10.239	10.239
5	15.938	13.819	12.558	12.109	11.948	11.927	11.927
10	18.491	15.650	13.916	13.273	13.094	13.054	13.054
100	38.174	28.286	21.456	19.031	18.138	17.908	17.908
10000	53.264	38.529	26.580	22.465	20.602	20.309	20.252
+∞(C-C)	53.474	38.692	26.662	22.514	20.932	20.347	20.274
Lei [22]	20.112						

Where *χ* is the weight coefficient, which is determined by the corresponding boundary conditions.

### In-plane load distribution and critical mode shapes

**Fig 3:**
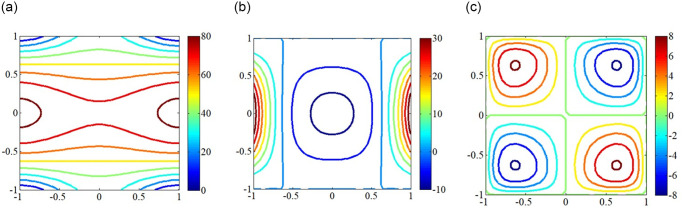
In-plane load distribution for SSSS edged square plate under subjected to convex edge loading having different directions. (a) *N*_*x*_; (b) *N*_*y*_; (c) *N*_*xy*_.

**Fig 4:**
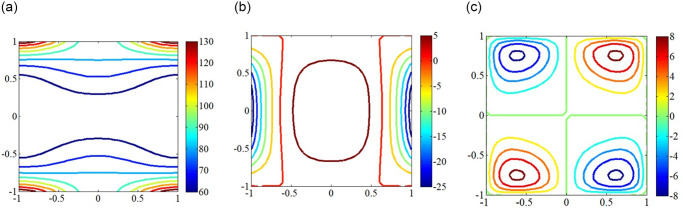
In-plane load distribution for SSSS edged square plate under subjected to concave edge loading having different directions. (a) *N*_*x*_; (b) *N*_*y*_; (c) *N*_*xy*_.

**Fig 5:**
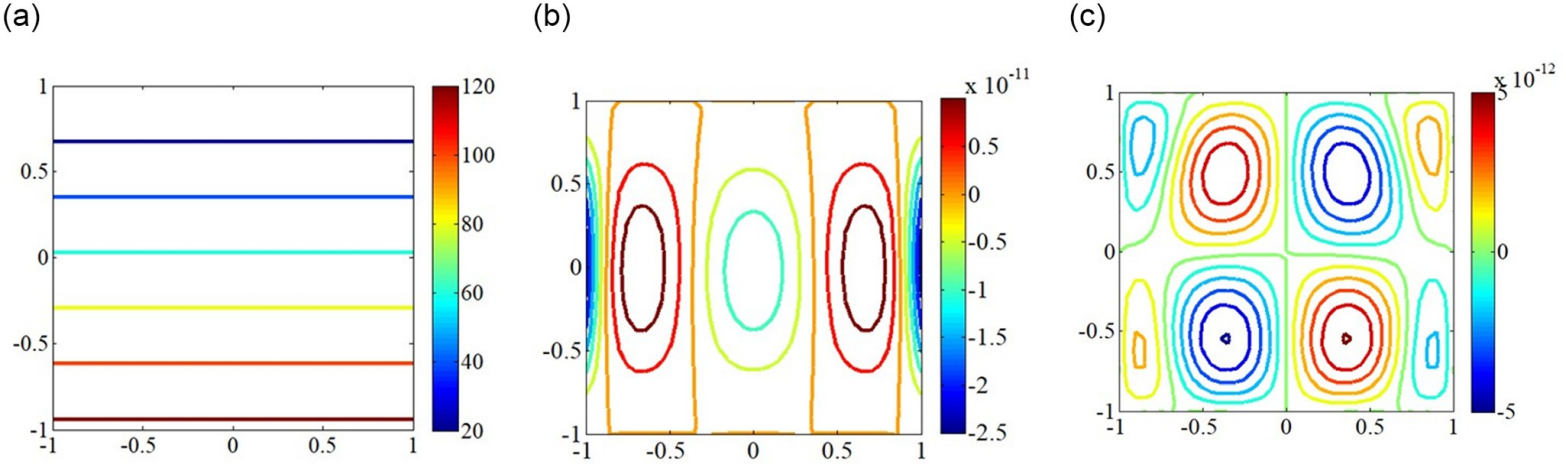
In-plane load distribution for SSSS edged square plate under subjected to trapezoidal loadinghaving different directions. (a) *N*_*x*_; (b) *N*_*y*_; (c) *N*_*xy*_.

**Fig 6:**
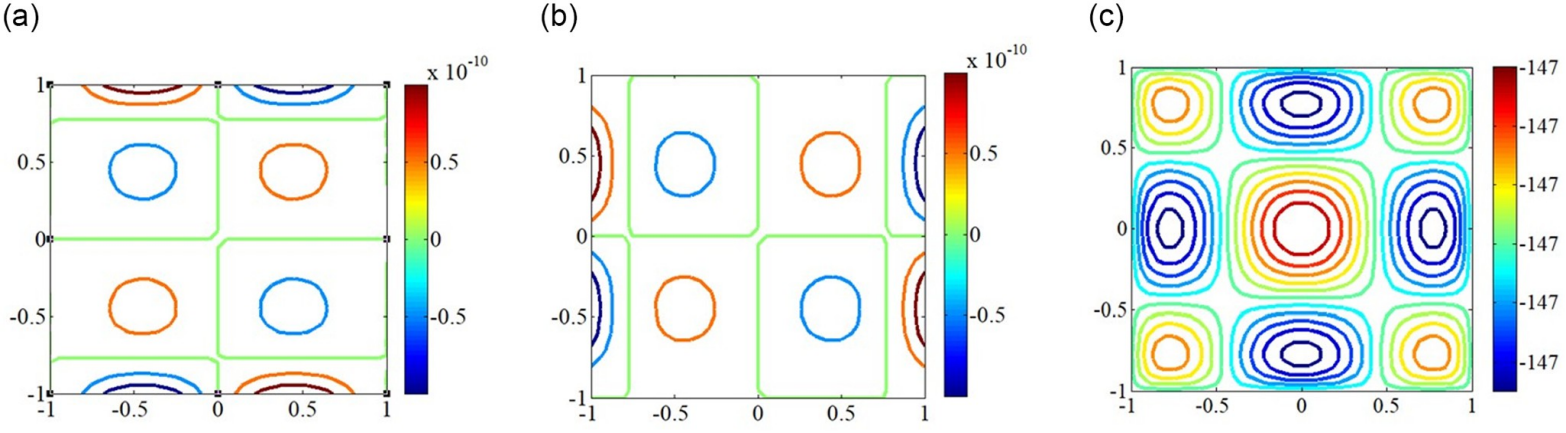
In-plane load distribution for SSSS edged square plate under subjected to shear loadinghaving different directions. (a) *N*_*x*_; (b) *N*_*y*_; (c) *N*_*xy*_.

The in-plane load distribution plays significant role on the buckling behavior of plates, in which the state of in-plane load distribution is depends on the parameters like skew angles, load types, boundary condition, etc. Plates with elastically restrained edges under non-uniform in-plane loading may cause non-uniform in-plane load distribution, which may lead to changes in critical buckling load of plates. Hence, it is highly necessary to study the in-plane load distribution and mode shapes for plates under non-uniform in-plane loading. It is observed from Figs 3–6 that the in-plane load extends from the load center towards the surrounding areas, in which the in-plane load is symmetrically distributed along the y-axis. Besides that, the more concentrated the in-plane load is at the boundary, the greater the critical buckling load. At the same time, the region of the in-plane load distribution gradually decreases, coupled with the increase in load intensity results in increase of critical buckling load.

**Fig 7:**
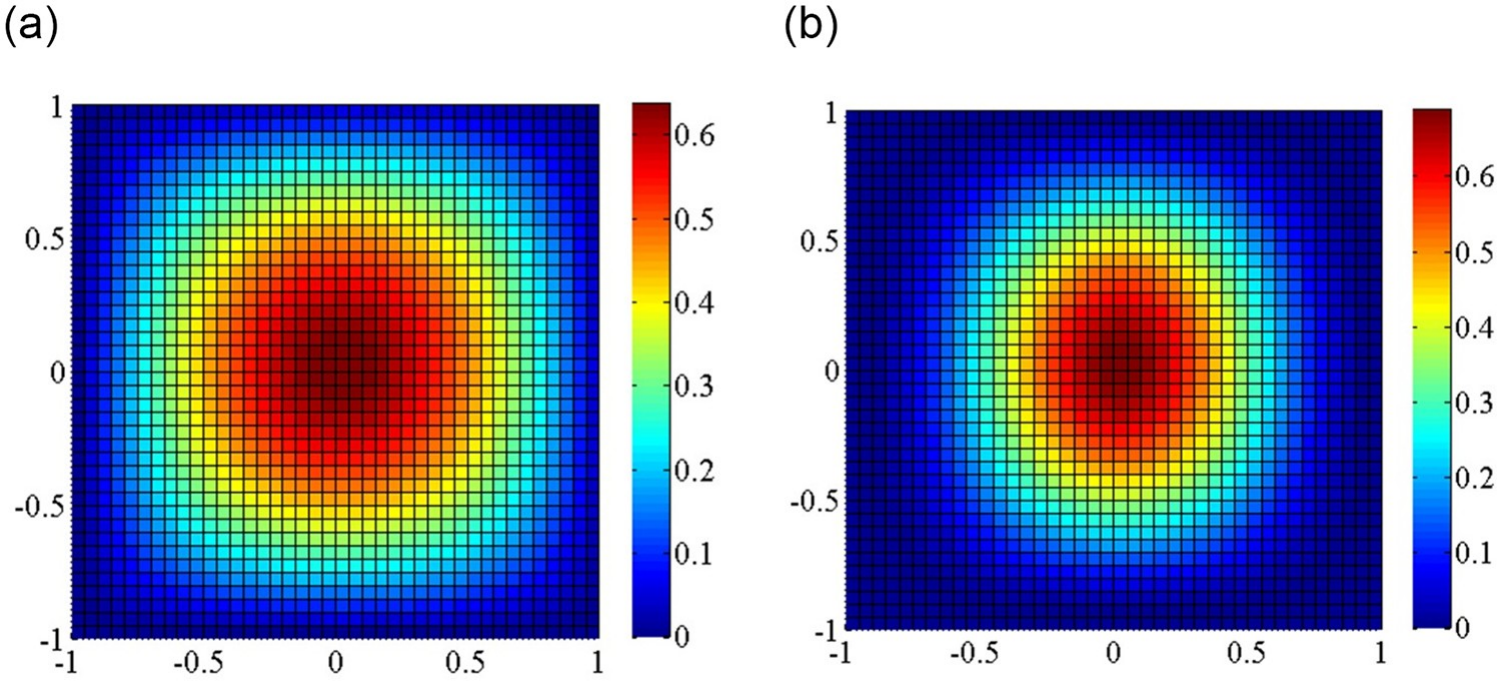
Critical mode shapes for the skew plate with different boundary conditions under axial edge load. (a) Simply supported boundary; (b) Clamped supported boundary.

**Fig 8:**
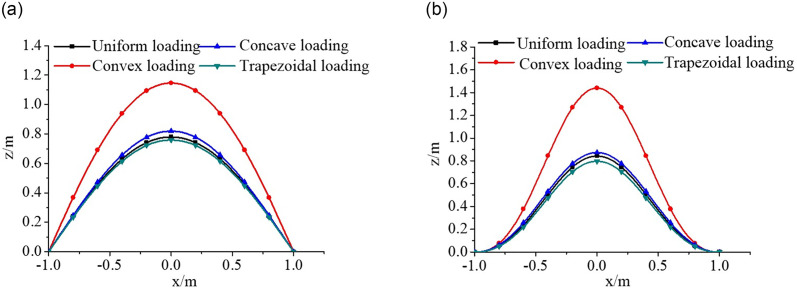
Cross-section curves of critical mode shapes under different non-uniform edge loadings. (a) Simply supported boundary; (b) Clamped supported boundary.

In addition, Figs 7 and 8 also provides the first buckling mode shape of the plate subjected to different non-uniform edge loadings. As can be seen that the half wave number of the buckling mode shapes under different non-uniform edge loadings remains the same. When the edge loading is concentrated near the central axis of the plate, the maximum deformation of plates occurs around the central part of the plate, which leads to a decrease in the bending resistance of the plate as the stiffness of the plate is very less in that portion. Therefore, the mode shape for any configuration depends not only the edge load type but also on its boundary condition.

**Fig 9:**
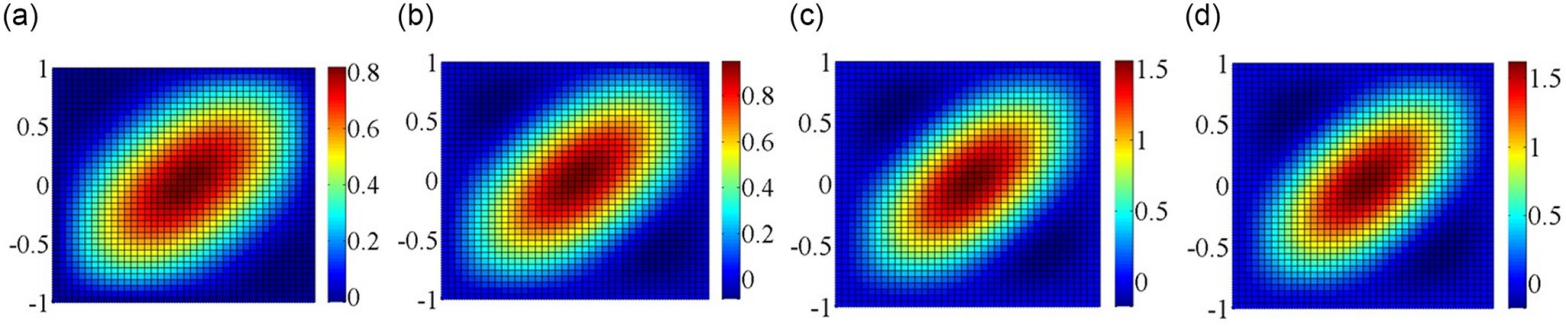
Critical mode shapes for the plate with different edge spring stiffnesses under shear edge loading. (a) *kx* = *ky* = 0; (b) *kx* = *ky* = 10; (c) *kx* = *ky* = 100; (d) *kx* = *ky* = 10000.

The buckling mode shapes of plates with elastically restrained edges under shear loads are shown in Fig 9. The high protruding area gradually decreases with the increasing the magnitude of the edge spring stiffness (k = 0, 10, 100 and 10000) applied to all four edges of the plate. The half wave number of the buckling mode shapes remains the same for the plates with elastically restrained edges. Besides that, as the increase of the edge spring stiffness, the radius in the central top region decreases which leads to an increase in critical buckling load.

**Fig 10:**
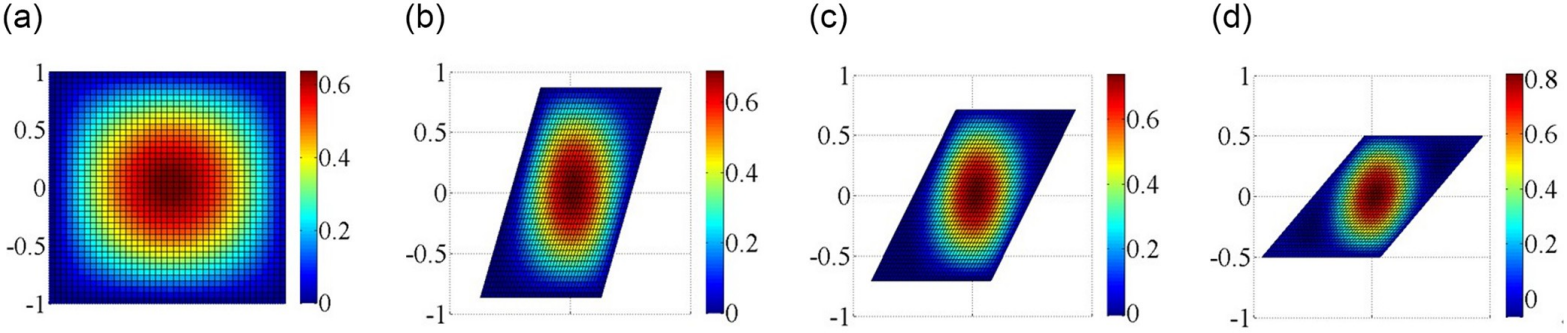
Critical mode shapes for the plate with different skew angles under boundary condition simply supported on four sides. (a) 0°; (b) 30°; (c) 45°; (d) 60°.

**Fig 11:**
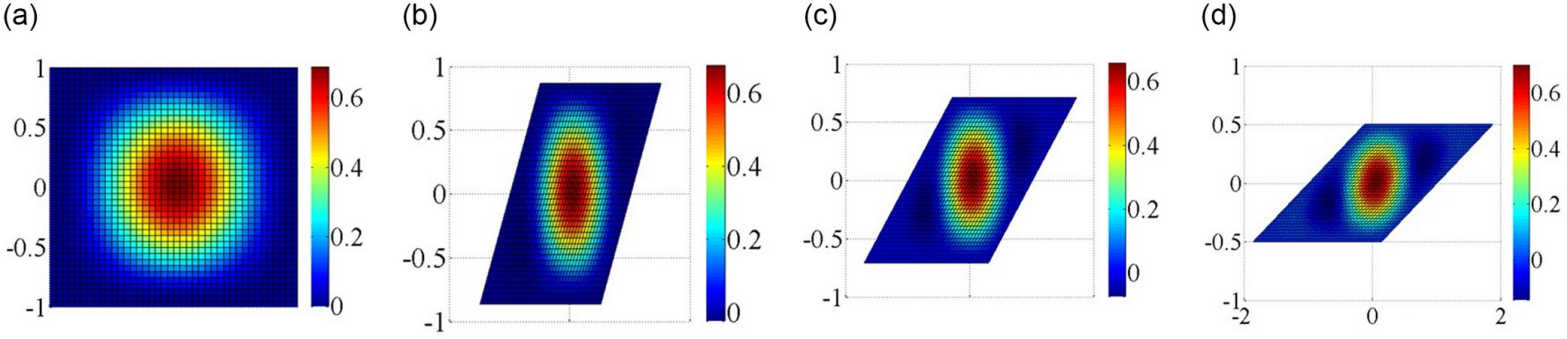
Critical mode shapes for the skew plate with different skew angles under boundary condition clamped supported on four sides. (a) 0°; (b) 30°; (c) 45°; (d) 60°.

Consider next the buckling of SSSS and CCCC skew plates under axial loadings. Four skew angles are investigated in Figs 10 and 11. In the skew plates, the center region is bulged out, while the free edges of skew plates bulge in the opposite directions. The half wave number of the buckling mode shapes remains the same for the skew plates with four different skew angles. The rate of increase in strength is more pronounced at higher skew angles.

### Parametric studies

In this section, the parametric analysis of buckling behaviour of the plates with respect to the edge spring stiffness, non-uniform edge loading, skew angle, aspect ratio and combined compression-shear load is presented and discussed in the following sections.

#### Effect of non-uniform edge loading on buckling behaviour of rectangular plates

**Fig 12:**
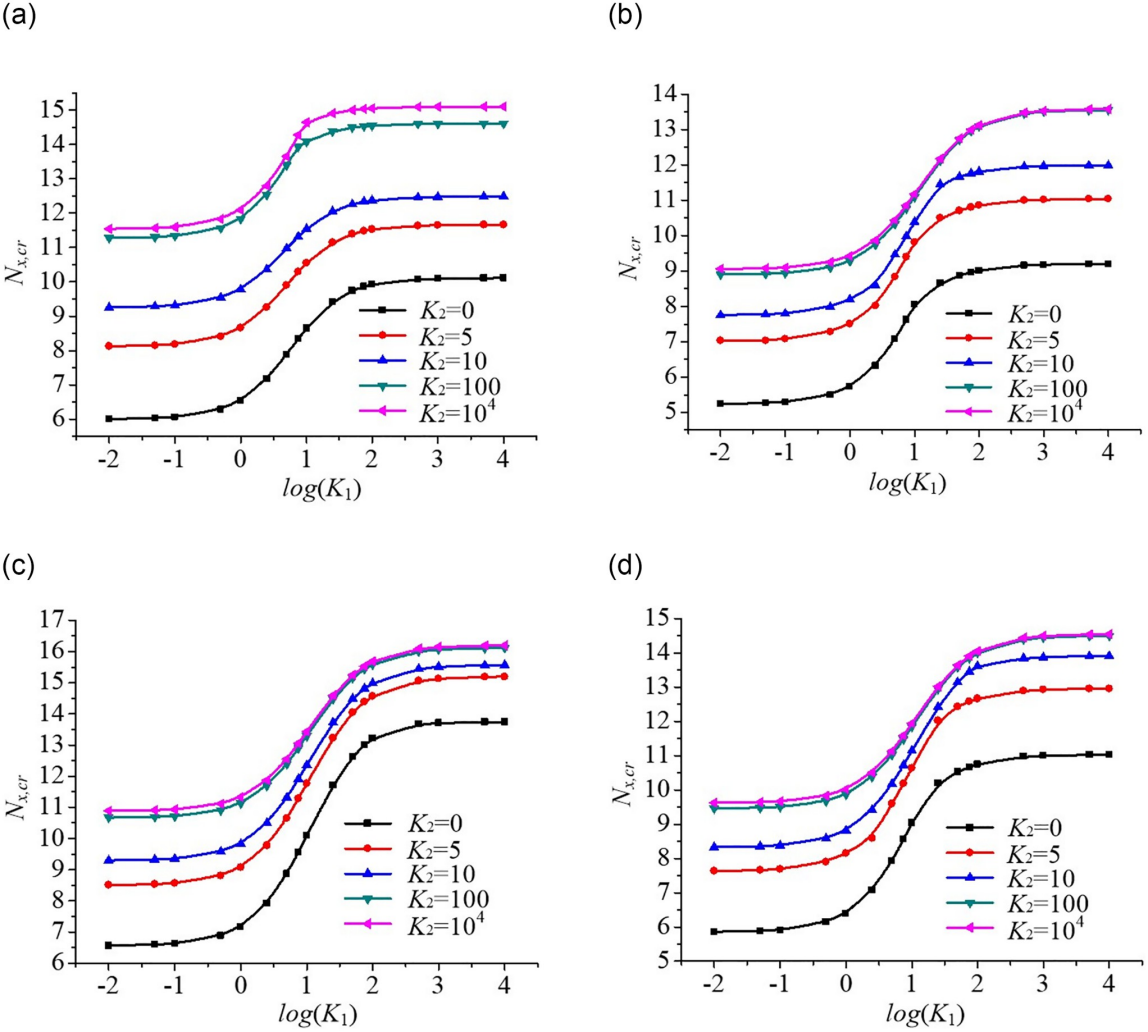
Dimensionless compressive buckling load *N*_*x*,*cr*_ vs. edge spring stiffness coefficients (*K*_1_ and *K*_2_) for rectangular plates under different axial edge loadings. (a). Uniform loading. (b). Parabolic loading (convex function). (c). Parabolic loading (concave function) (d). Trapezoidal loading.

The critical buckling loads of rectangular plates are plotted against the base 10 logarithm of the longitudinal edge spring stiffness coefficient *K*_1_ as shown in Fig 12. The obtained results show the critical buckling loads gradually increase with increasing transverse edge spring stiffness coefficient *K*_2_, in which the critical buckling load is the minimum for Parabolic loading (convex function), and maximum for Parabolic loading (concave function) at the same edge spring stiffness. Furthermore, the range of action of the edge spring stiffness coefficient on the buckling analysis of the rectangular plate under the non-uniform mechanical edge loading is smaller than that under the uniform mechanical edge loading and the general trend in the buckling loads of the plate is consistent under various axial edge loading considered. However, the rate of increase of critical buckling loads under non-uniform mechanical edge loading is relatively large.

#### Effect of combined compression-shear load on buckling behaviour of rectangular plates

**Fig 13:**
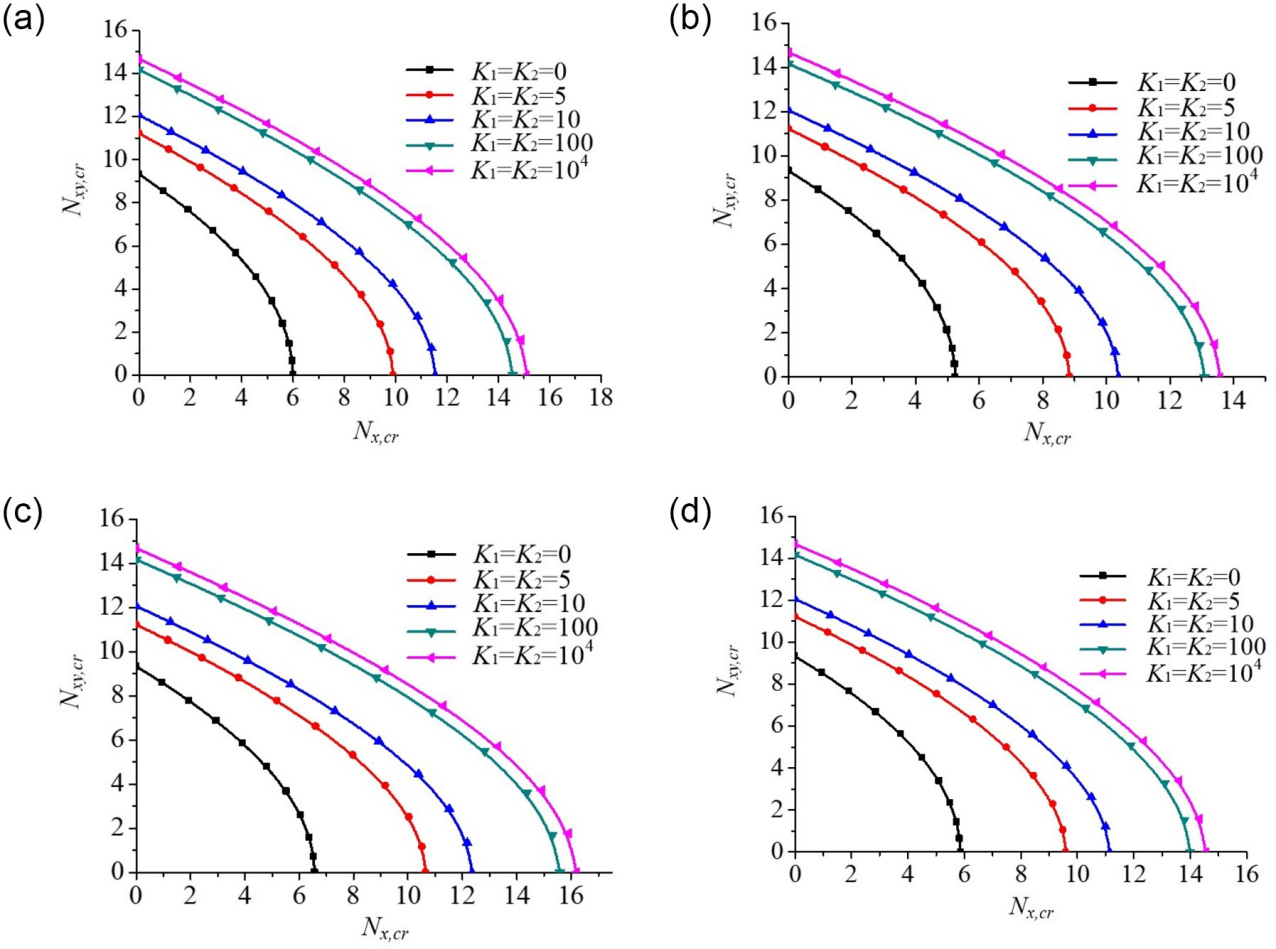
Dimensionless compressive buckling load *N*_*x*,*cr*_ vs. edge spring stiffness coefficients (*K*_1_ and *K*_2_) for rectangular plates under combined compression-shear load. (a). Uniform loading. (b). Parabolic loading (convex function). (c). Parabolic loading (concave function). (d). Trapezoidal loading.

A parametric analysis is performed to evaluate the effect of combined compression-shear load (*N*_*xy*_ / *N*_*x*_) on the buckling behaviour of rectangular plates as shown in Fig 13. The critical shear buckling loads of the plate with five edge spring stiffness coefficients (*K*_1_ = *K*_2_ = 0, 5, 10, 100, 10^4^) are calculated under different axial compressive edge loadings. It can be seen that the compressive buckling loads decrease gradually with increasing shear buckling loads. This can be explained by the fact that stress distribution is negated by the shear loading. Furthermore, the absolute values of the slopes of the buckling interaction curves for the plate gradually increase as the axial compressive edge loading increases, in which the absolute value of the slope of the buckling interaction curves is the maximum for Parabolic loading (convex function), and minimum for Parabolic loading (concave function) when the value *N*_*xy*_ / *N*_*x*_ is the constant.

#### Effect of aspect ratio on buckling behaviour of rectangular plates

**Fig 14:**
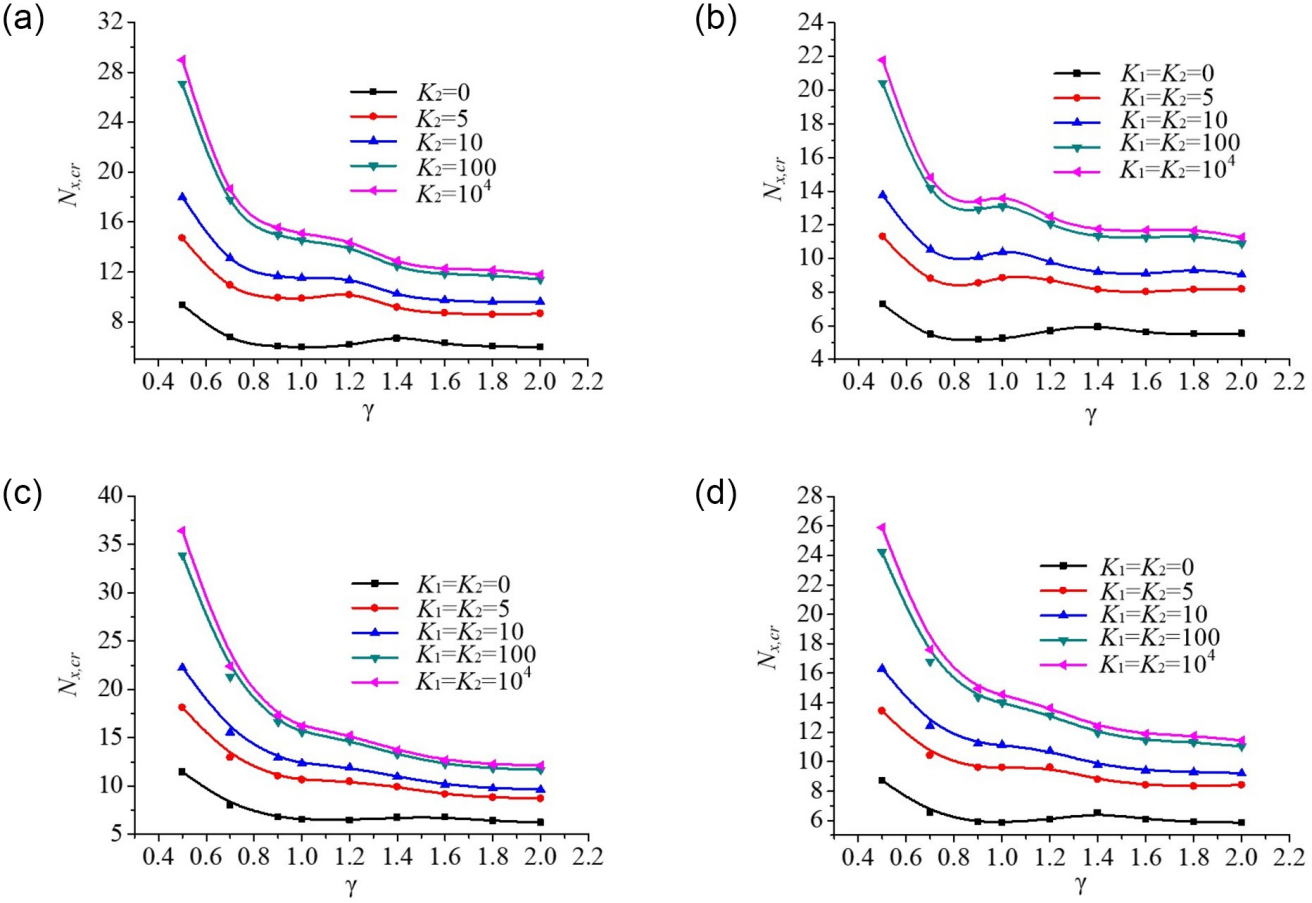
Dimensionless compressive buckling load *N*_*x*,*cr*_ vs. aspect ratio γ for rectangular plates under different axial edge loadings. (a). Uniform loading. (b). Parabolic loading (convex function). (c). Parabolic loading (Concave function). (d). Trapezoidal loading.

The effect of aspect ratio *γ* = *a*/*b* on the buckling behaviour of rectangular plates under non-uniform mechanical edge loading is shown in Fig 14. The critical buckling loads of the plate are plotted against plate aspect ratio for five edge spring stiffness coefficients. The results show that the rate of critical buckling loads of the plate is more sensitive when the aspect ratio *γ* is in the range of 0.5–1; conversely, there is almost no change when the aspect ratio is in the range of 1–2. Besides, the critical buckling loads gradually decrease with increasing plate aspect ratio and decreasing edge spring stiffness. That’s because, the higher the plate aspect ratio *γ*, the smaller the plate’s stiffness. Furthermore, the critical buckling loads of the plate increase in the following order: Parabolic loading (convex function) < Trapezoidal loading < Uniform loading < Parabolic loading (concave function) at the same plate aspect ratio. Particularly the rate of decrease of critical buckling load is the maximum for the aspect ratio *γ* = 0.5.

#### Effect of edge spring stiffness on buckling behaviour of skew plates

**Fig 15:**
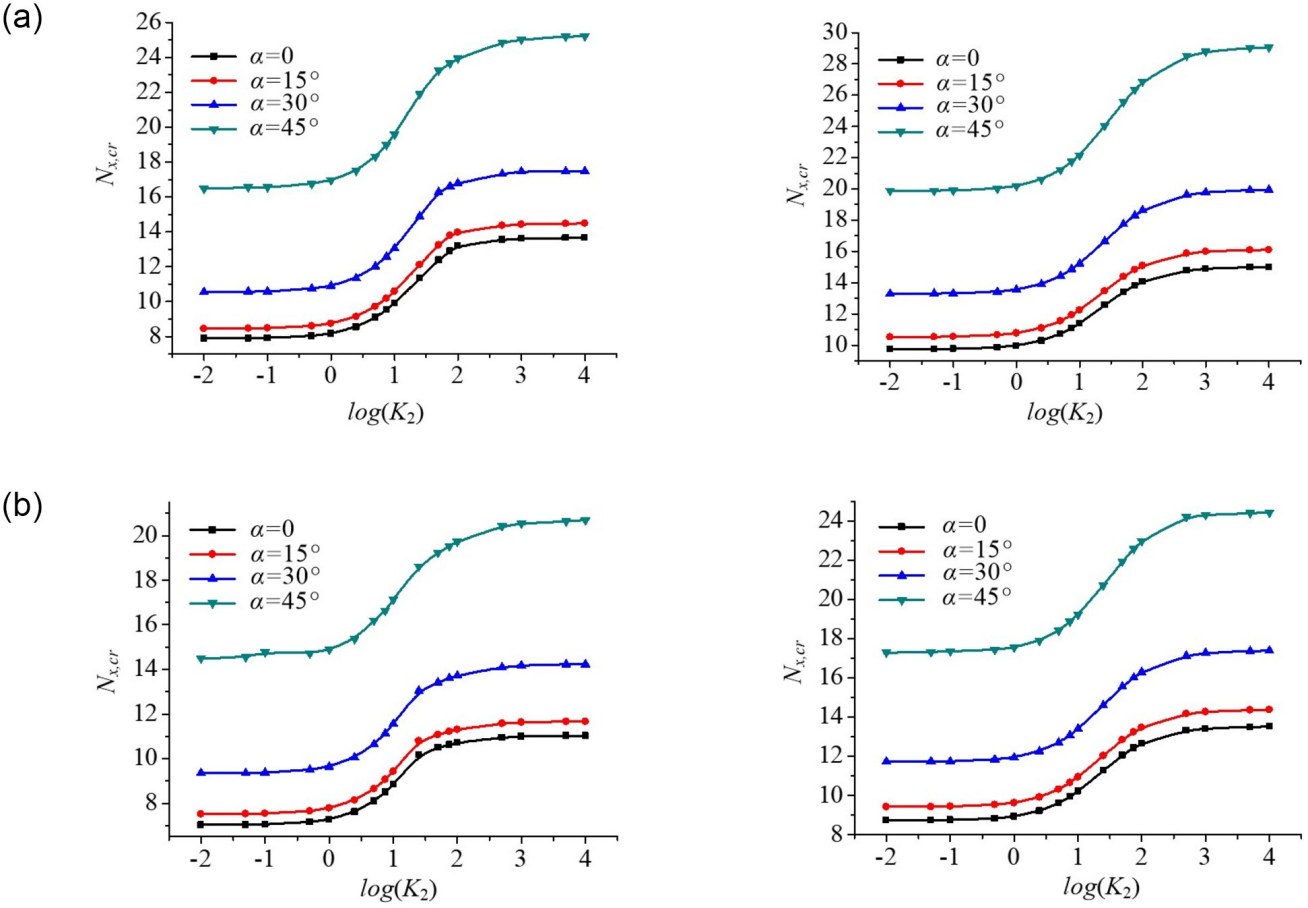
Dimensionless compressive buckling load *N*_*x*,*cr*_ vs. edge spring stiffness coefficients (*K*_1_ and *K*_2_) for skew plates under different axial edge loadings. (a). Uniform loading (*K*_1_ = 10,100). (b). Parabolic loading (*K*_1_ = 10,100).

Fig 15 shows that the critical buckling loads of the skew plate are plotted against the base 10 logarithm of the loaded edge spring stiffness coefficient *K*_2_ for different axial edge loadings, in which skew angle is in the range of 0°-45°. The critical buckling loads of skew plates with unloaded edge spring stiffness coefficients (*K*_1_ = 10,100) are calculated. It is observed that the critical buckling loads increase with the increasing loaded edge spring stiffness coefficient *K*_2_. Besides, the critical buckling loads also increase with the increasing skew angle *α*, in which the larger the plate skew angle *α*, the more sensitive the variation rate of critical buckling load. The critical buckling loads of skew plates are proportional to edge restrained spring stiffness.

#### Effect of plate aspect ratio on buckling behaviour of skew plates

**Fig 16:**
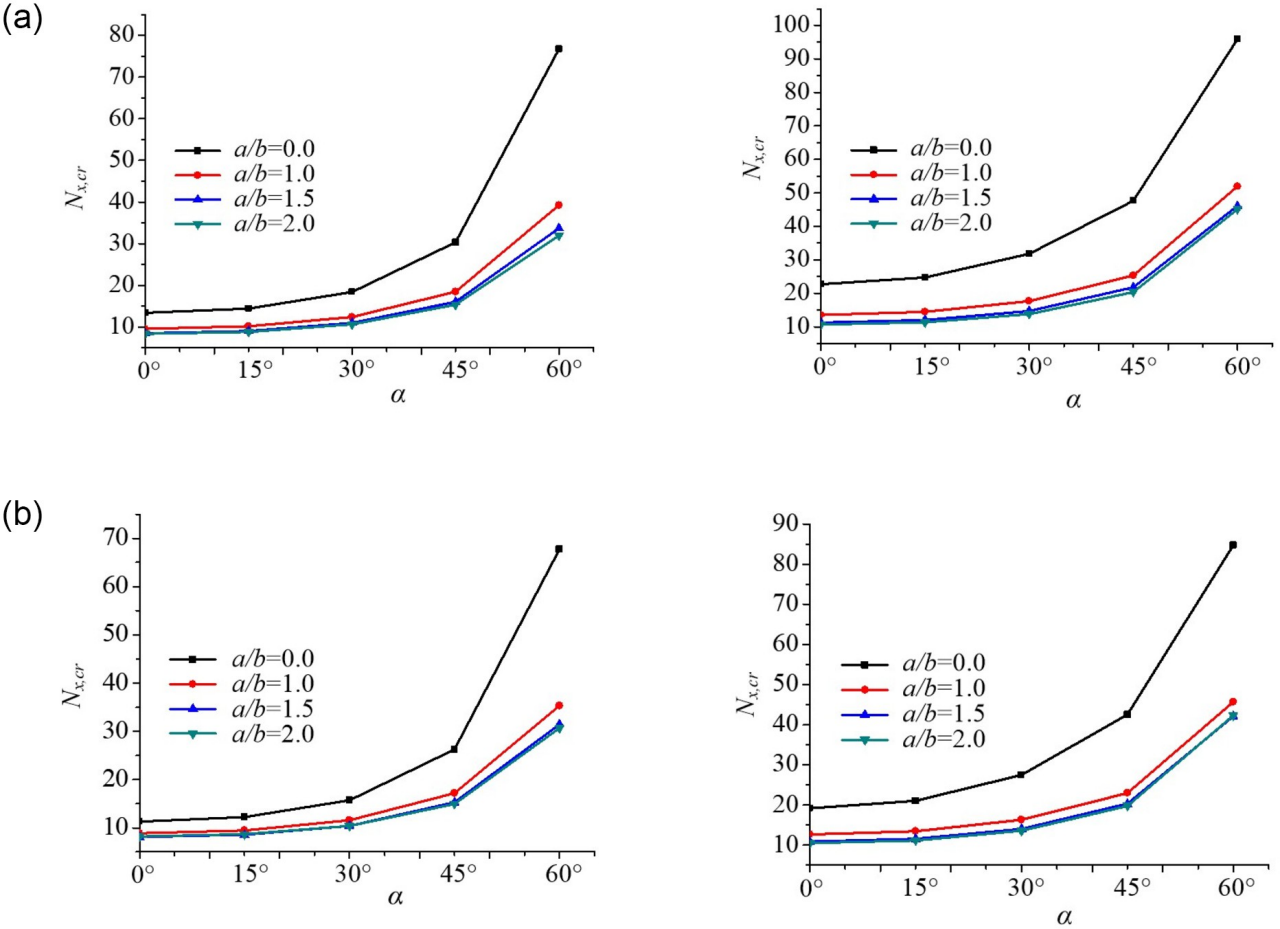
Dimensionless compressive buckling load *N*_*x*,*cr*_ vs. aspect ratio *γ* for skew plates under different axial edge loadings. (a). Uniform edge loading (*K*_1_ = *K*_2_ = 10,100). (b). Parabolic edge loading (*K*_1_ = *K*_2_ = 10,100).

Fig 16 presents the effect of the plate aspect ratios *γ* = *a*/*b* on the buckling behaviour of skew plates under uniform and non-uniform mechanical edge loading. The critical buckling loads of skew plates with two edge spring stiffnesses (*K*_1_ = *K*_2_ = 10,100) are investigated under different axial edge loadings. It can be seen that the critical buckling loads gradually increase with the increasing skew angles *α*, and the critical buckling loads decrease with the increasing plate aspect ratios. Besides, the critical buckling loads of the plate increase in the following order: Parabolic edge loading < Uniform edge loading at the same plate aspect ratio *γ*. The variation rate of the critical buckling loads of the skew plate is more sensitive when the plate aspect ratio is more than 1.5, conversely, there is almost no change when the aspect ratio is in the range of 0.5~1.5. Meanwhile, the rate of decrease of critical buckling load is the maximum for the aspect ratio *γ* = 2. The results show that buckling loading *N*_*x*,*cr*_ approximately increases by 2 and 5 times when *α* varies from 30° to 60°, respectively.

#### Effect of the skew angle on buckling behaviour of skew plates

**Fig 17:**
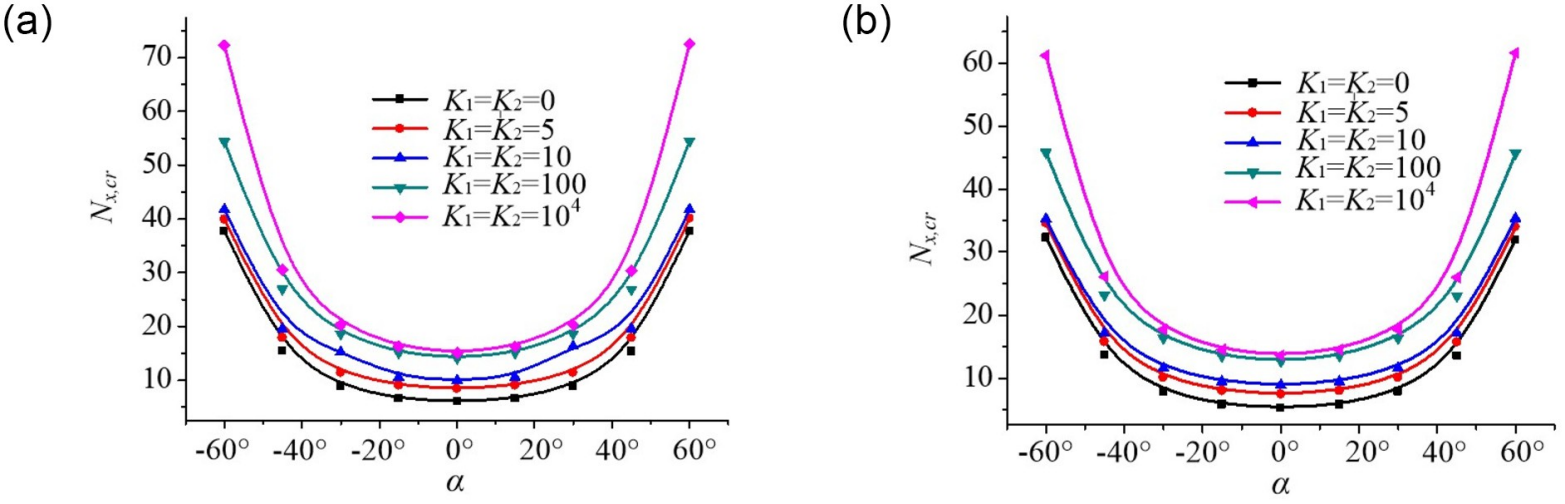
Dimensionless compressive buckling load *N*_*x*,*cr*_ vs. skew angle *α* for skew plates under different axial edge loadings. (a). Uniform edge loading. (b). Parabolic edge loading.

The effect of the skew angles on the buckling behaviour of the plates under uniform and non-uniform mechanical edge loading is shown in Fig 17, in which the critical buckling loads of skew plates are plotted against the skew angles of plates for five different edge spring stiffness coefficients *K*_1_ = *K*_2_ = 0,5,10,100,10^4^. It is observed that the critical buckling loads decrease first and then increases with the increasing skew angles. The critical buckling loads of plates increase in the following order: Parabolic edge loading < Uniform edge loading at the same edge spring stiffness. The variation rate of the critical buckling loads of the plate is more pronounced when the skew angles *α* is less than −30°, or greater than 30°, conversely, there is almost no change when the skew angles are in the range of −30°–30°. These results clearly indicate that the stiffness of the skew plate increases with the increase of skew angles at the same edge spring stiffness. In other words, a significant increase in buckling load occurs due to the enlargement of flexural stiffness of the plate.

## Conclusions

In this paper, the buckling behaviour of rectangular and skew plates with elastically restrained edges under different non-uniform edge loadings is investigated using the Ritz method. A proposed method with Legendre polynomials is used to solve equilibrium differential equations of the plate. The in-plane stress distribution under non-uniform edge loading is defined by the pre-buckling analysis. The proposed analysis method is in good agreement with results from the literatures. The results show that the shape function and in-plane stress function subjected to different non-uniform edge loadings are effective for the buckling analysis of skew plates in this paper. A parametric analysis is conducted to evaluate the effects of the edge spring stiffness, non-uniform edge loading, skew angle, aspect ratio and combined compression-shear load on buckling behaviour of plates.

In summary, the proposed analysis method is capable of accurately predicting the buckling load of skew plates under non-uniform edge loading. It is found that: (1) the critical buckling loads gradually increase with the increasing edge restrained spring stiffness coefficients, in which the critical buckling load is the minimum for Parabolic loading (convex function), and maximum for Parabolic loading (concave function) at the same edge spring stiffness; (2) the critical buckling loads gradually decrease with increasing plate aspect ratio and decreasing edge restrained spring stiffness; (3) the critical buckling loads gradually increase with increasing skew angles *α*, and the critical buckling loads decrease with increasing plate aspect ratios; (4) the critical buckling loads decrease first and then increases with the increasing skew angles.

## Supporting information

S1 Data (XLSX)
